# The need for speed - adenosine stress MRI in less than 30 minutes

**DOI:** 10.1186/1532-429X-15-S1-E24

**Published:** 2013-01-30

**Authors:** Susan J Thomas, Mark G Bryant, Johannes Radvan, Peter Speier, Edgar Mueller, Michaela Schmidt, Carmel Hayes, Russell Bull

**Affiliations:** 1 Department of Radiology, Royal Bournemouth Hospital, Bournemouth, UK; 2 Department of Cardiology, Royal Bournemouth Hospital, Bournemouth, UK; 3 Siemens AG, Erlangen, Germany

## Background

Strong evidence in favour of an ischaemia-guided approach to coronary revascularisation for PCI has led to a huge increase in demand for ischaemia imaging. In our General Hospital setting we have seen a 300% increase in demand for stress MRI over 3 years to more than 800 examinations per year.

We therefore sought to optimise patient throughput by process mapping and by the introduction of new semi-automated scanning software. The aim of the study was to assess the impact of these changes on MRI study time.

## Methods

249 consecutive adenosine stress MRI studies from a period of 5 months were examined. Two cardiac optimized MRI scanners were used, (1.5T Siemens MAGNETOM Avanto) and (1.5T Siemens MAGNETOM Symphony). One scanner (Avanto) had additional novel cardiac scanning software (Siemens Cardiac DOT™), which provided a semi-automated approach to cardiac MRI scanning, with rapid planning of cardiac planes and automatic adjustment of imaging parameters depending on patient heart rate and breath-hold capability. The other scanner used conventional cardiac software. Both scanners were operated by the same group of general MRI radiographers and used the same scan sequences. In both, a stress only protocol was used with no rest images. Phase Sensitive Inversion Recovery (PSIR) late gadolinium enhancement images were acquired such that no TI (inversion time) test was required. The time taken from the initial planning scans to the end of the final sequence was measured in each of the 249 cases. Comparison was then made between the two scanners to assess the impact of the new software on study time.

## Results

Of the 249 stress scans, 214 (86%) were performed on the scanner with cardiac DOT software and 35 (14%) on the scanner with conventional software. The scan times for studies using the cardiac DOT software were significantly less, with scan times of 15.8 - 42.5 (mean 27.7) minutes vs. 26.4 - 58.4 (mean 41.1) minutes respectively (p<0.00001).

## Conclusions

The streamlined adenosine stress protocol alone led to a short mean scan time of 40 minutes. With the combination of a streamlined protocol and semi-automated software it was possible to consistently perform cardiac stress MRI with a mean scan time of 28 minutes. Wider adoption of this approach could have implications regarding cost savings and increased patient throughput.

## Funding

A lump sum of 10 000 euros was received by the Department of Radiology, Royal Bournemouth Hospital from Siemens Healthcare for developing the Siemens cardiac DOT engine.

